# The patient perspective: investigating patient empowerment enablers and barriers within the oncological care process

**DOI:** 10.3332/ecancer.2019.912

**Published:** 2019-03-28

**Authors:** Luca Bailo, Paolo Guiddi, Laura Vergani, Giulia Marton, Gabriella Pravettoni

**Affiliations:** 1 Applied Research Unit for Cognitive and Psychological Science, European Institute of Oncology, 20141 Milan, Italy; 2 Department of Oncology and Hemato-Oncology, University of Milan, 20122 Milan, Italy

**Keywords:** patient empowerment, quality of healthcare, patient participation

## Abstract

Patient empowerment is a multi-factorial concept and its relevance has led to a growing body of literature; despite this attention, there is still no agreement regarding the elements that define its expression. While several studies have already investigated the positive effect of empowerment interventions on the care process outcome, the aim of this study is to investigate which factors can foster an empowered management of the cancer condition from the patient’s perspective. To examine patients’ perception of empowerment enablers, we asked for participants’ input on the role of three factors frequently cited as positively affected by empowerment: care quality, perception of direct control and relationships within the care context, during the care process. Three focus groups were conducted with 34 cancer patients. The results highlight the perception of direct control on their treatment as the least valued element (2.87, SD 0.566) when compared with care quality (3.75, SD 0.649) and relational support in the care context (3.91, SD 0.274).

Unlike traditional approaches to empowerment, patient’s expression of empowerment does not mainly reside in the direct control of their condition as much as in an active role within the relationship with caretakers, such as the ability to choose the doctor, the care team or the health organisation in charge of their healthcare. Emerging aspects from this analysis of patient’s perspective are central in order to adequately consider empowerment in the care process and to provide more effective care strategies.

## Background

In the last decades, empowerment has gained importance in health care and its relevance has led to a growing body of literature [1]. Even though the majority of authors agree about the importance of patient empowerment in the healthcare context, there is no unique definition of this construct. In September 2012, the World Health Organization proposed to define empowerment as ‘a process through which people gain greater control over decisions and actions affecting their health’ which can be obtained through skill development, access to information and resources and influencing those factors that affect their health and well-being [2].

However, the complexity of patient empowerment and the wide scope of its effects across the care process induced several authors to investigate the different dimensions of this construct and to develop different ways to define and describe its components. A recent review [3] of patient empowerment found 17 different definitions and described ten possible dimensions in which it may be expressed, such as participation in clinical decision-making, patients’ control over their condition and knowledge acquisition. These elements showed that empowerment can be framed both as an outcome and as a process: as a process, by considering all those elements that can influence the participation in the different phases of the care process and shift the decisional power towards the patient; as an outcome, by focusing on the results of this development. In other words, this means that empowerment may be seen as an enabling process in which healthcare professionals cooperate with patients to help them acquire knowledge and resources; its outcome is a patient with a greater ability to manage his/her condition and to make informed decisions (ibidem).

The empowerment process, and the expression of all its outcomes, is not something that depends solely on patient’s characteristics and strategies. The relationship with the context in which the patient faces cancer has a fundamental influence on the empowerment process and allows its representation as a tripartite construct [4] with three dimensions: intrapersonal, interactional and behavioural. The first component takes into account how people perceive themselves and represents their competence and ability to control the management of their condition. The second one considers the environment that patients have to deal with during the care process. The behavioural component, finally, describes actions taken to exert control over what is happening to them. Within this framework, in order to be empowered, patients need to achieve ‘a critical awareness of the environment and an active engagement in it’ [5]. Since patients do not have direct control over their condition, they need to be actively engaged to access different information sources and to relate with people involved in the care process, from caregivers, who provide support inside and outside of the hospital context, to healthcare professionals, who are in charge of the clinical treatment.

Instead of focusing on the effects that empowerment has on the patient’s condition, both from a clinical and psychological perspective, we aim to investigate what patients perceive as empowerment enablers from their perspective. In other words, we are going to focus on those elements that patients see as helpful in managing their condition, instead of observing the effect that empowerment-oriented interventions has on the care process.

## The cancer patient perspective

Due to the evolution of treatments and the increase in survival rate of patients, cancer is currently considered one of the most common chronic diseases [6]. Hence, the empowerment of cancer patients has gained considerable attention for its effect on their treatment [7–11].

Empowerment in patients with chronic diseases has shown several positive effects, such as increased patient satisfaction with care, improved patient adherence to self-management of the treatment and better clinical outcomes [12–15]. Since several authors have already addressed the effect that empowerment may have on the care process, to have an integrated view on this concept, we investigated which elements may influence the development of empowerment from the patient’s perspective.

In order to investigate the elements that allow patients to efficiently manage their condition, we focused on three dimensions that are highly relevant in several framings of empowerment: the patients’ perception of the quality of care, the control they may exert over the care process and their relationship with healthcare providers and informal caregivers [54]. The perceived quality of care is one of the areas that patients value as responsible for their empowerment. Quality of care is a complex concept that can be evaluated both by quantitative elements, such as mortality rates and reduction in adverse events, and qualitative measures, such as patients’ general satisfaction with the care process [16]. The adoption of empowerment interventions in the care context showed a direct effect on the quality of care as perceived by oncological patients undergoing surgery on clinical outcomes, such as postoperative pain [17]. Moreover, several studies [18–20] also reported that empowering healthcare professionals has a positive effect on the quality of care and, consequently, on the patients’ reported satisfaction [21].

Still, elements other than clinical outcomes have an impact on the perceived quality of the care process. An important element that patients value when assessing care quality is the healthcare providers’ ability to communicate clinical information [22]. Since knowledge gathering is a crucial skill to empower patients, their access to information is strongly related to the ability of healthcare professionals to provide and explain knowledge [23, 24]. The ability to manage their condition necessarily starts from the awareness that patients acquire both about their condition and about the consequences of different treatment options.

The second element frequently associated with patients’ empowerment is their ability to exert control over their condition. From this perspective, it can be framed as the ability to ‘gain greater control over decisions and actions affecting their health’ [25]. From the patient perspective, control is both an outcome of the empowerment process and an enabling factor that they perceive as fundamental in order to feel empowered and have an active role in their care process [26]. In other words, this means that empowered patients have more control over their condition and, on the other hand, being able to exert control over their condition enables them to be more empowered. The most common expression of control considered in the literature is the ability to make decisions about the treatment they are undergoing, such as the choice between different treatment options. However, patient’s control may also be expressed in several other ways, such as their self-management after hospitalisation. In this context, patients have full control over symptoms monitoring and self-administration of pharmacological therapy, fundamental in the treatment of chronic diseases and for cancer survivors.

One of the less considered forms of control over their care process is the patients’ ability to choose which physician and hospital to go to in order to receive care [27, 28]. From this perspective, patients’ expression of control affects specific clinical decisions and also broader logistical issues that may steer their preference in the selection of the care provider and, hence, may change their treatment entirely.

Finally, empowerment may also be defined as ‘a process and outcome arising from communication between healthcare professionals and patients’ [26]. In recent years, the relationship between patients and physicians has become increasingly important [3], focusing on the benefits of communication and cooperation with professionals in the healthcare context [15, 26, 29–32]. This interpersonal dimension of empowerment [33] considers communication, education and the sharing of values, knowledge and power as fundamental components of the patient–clinician relationship which fosters the process of empowerment.

Several authors [22] highlight the risk of misunderstanding the relational value of empowerment. Often, an improved compliance and adherence to the care plan are expected consequences of patient empowerment; sometimes, however, the latter can be considered the antithesis of the first two (ibidem). Believing that a more informed patient will agree with the clinician just because he can better understand the medical reasoning underlying the decision-making process does not take into consideration that the patient’s priorities and preferences may not be the same as the clinician’s. In this context, the empowerment-based approach on medical decision making provides a radically different perspective when compared with the traditional approach based on compliance [33]. While in the latter approach, the patient’s role is to be a ‘passive recipient of care’ [34], the first approach considers patients as an active part of the care process, sharing responsibilities with healthcare professionals for the outcome of their treatment [33].

We recognised three main areas (patients’ perception of the quality of care, patients’ control over the care process and relationship with healthcare providers and informal caregivers) as the ones that mainly rely on the patients’ perspective. We believe that information on these areas, as reported by patients themselves, can foster a deeper understanding of those elements that can actually empower patients in the management of their condition.

One potential technique for exploring quality of life (QoL) concerns is the use of focus group methodology. Focus groups have been described as a useful yet underutilised research technique in health care research, yet long used in business and industry [45, 46]. Focus group interviews or discussions have proven to be quite effective in obtaining perceptions on a defined area of interest in a permissive, nonthreatening environment [47, 48]. Howard *et al*. [49] defined focus groups as ‘a discussion in which a small group of people…under the guidance of a moderator facilitator, discuss topics selected for investigation.’ Basch’s [50] definition of focus groups is ‘a qualitative research technique used to obtain data about feelings and opinions of small groups of participants about a given problem, experience, service or other phenomenon’. Focus groups have been utilised in both clinical and research settings. The impetus for using focus groups in the clinical setting has been on improving practice and quality care through consumer input [51, 52]. Several examples of the use of focus groups in research can be identified in the literature.

## Methods

The conceptual framework for this study relies on the empowerment model for cancer based on the analytical themes identified in the literature and the recent review by Jorgensen *et al*. [35].

The main aim of this study is to investigate (1) the importance of the three main elements we provided in the patient’s management of their condition and (2) how those elements can foster patient empowerment.

### Participants

We conducted three separate focus groups with 34 cancer patients followed by European Institute of Oncology (IEO) clinicians. Both day hospital and hospitalised patients were considered eligible to participate.

A convenience sample was derived from review of records and physician’s/nurse’s referrals. All participants were recruited at the IEO, Milan, Italy, where we also conducted the focus groups. The inclusion criteria required to be enlisted for focus group sessions were to be (1) a cancer patient, (2) an adult (+18 years/old), (3) currently listed as a patient of the IEO (Table 1). Since we aimed to investigate patient empowerment components, we decided to explore their perception on average without sampling for a specific condition (e.g., breast cancer).

### Psychologists of the psycho-oncological division informed eligible patients during the psychological support process

Those who decided to participate were contacted by phone and randomly assigned to one of the scheduled focus groups. Participants provided signed informed consent and completed a brief initial demographic survey.

## Focus group procedure

The focus group [36] sessions were conducted in the IEO centre and facilitated by two psychologists.

A moderator trained in qualitative research methods conducted the focus groups using a semi-structured interview as a guideline. Participants were introduced to the concept of empowerment as defined by the literature and were informed of the main topics of the session (quality of care, direct control over their treatment and the relationship with the healthcare professionals) as elements of support in dealing with the disease condition. In order to clarify and foster an open discussion, we firstly asked them to grade those elements from 1 to 5, and, from their feedback, we prompted a discussion for each topic of the session.

Questions were designed to explore the experiences of patients regarding these main topics at the time of diagnosis and during treatments. The interview guide format allowed the moderator to use probes to explore participants’ responses further while maintaining consistency across the focus groups [37].

During the focus groups, participants were prompted to express their opinion about these areas and to provide new components that they valued as fundamental in the management of their current condition.

At the end of the focus groups, the moderator summarised major discussion points between questions, which provided participants with an opportunity to share any final thoughts and to clarify anything they felt was not correctly understood by the moderator. Focus groups lasted approximately 90 minutes each. Study staff took handwritten notes during each group and sessions were audio recorded for transcription.

### Analysis

The focus group audio recordings were transcribed with all identifiers removed. Subsequently, the authors read the transcription and selected meaningful text extracts in order to recognise the main themes addressed, using steps outlined by Strauss and Corbin [38]. First, a codebook was developed by examining the transcripts to identify themes and create initial coding categories. Second, all the authors examined quotes and decided to allocate each reported text piece as informative for a specific theme. Finally, initial codes were classified into broader categories and examined for emerging themes [39].

## Results

We conducted three focus groups with 10, 12 and 12 participants, respectively. Participants’ mean age was 54 (range: 30–89; SD: 12.57). The vast majority of participants were female (97.06%), with the exception of one single male patient: this was due to a high participation of patients from the breast and gynaecology cancer units. Participants were cancer patients suffering from breast (21, 61.8%), uterine (5, 14.7%), ovarian (4, 11.8%), urogenital (3, 8.8%) cancer and sarcoma (1, 2.9%).

Aim 1: What is the value given to different empowerment components?

**Figure 1.**
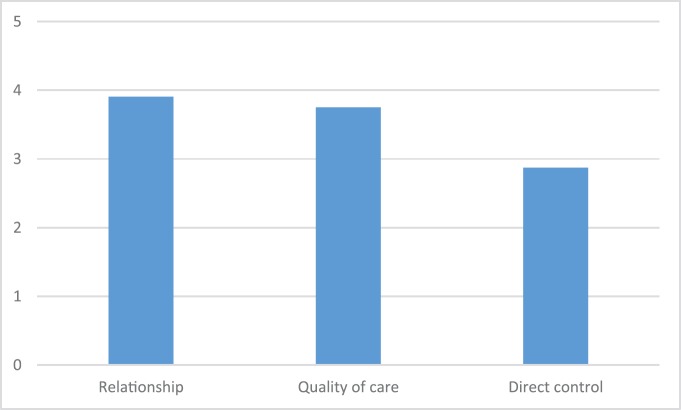
Patients’ evaluation of different elements that lead to empowerment.

We asked participants to rate from 1 to 5 the importance in the management of their condition of the three elements we proposed: Care quality, perception of direct control, relationships within the care context (Figure 1). The results allowed us to observe that the perception of direct control over their treatment was, by far, the least valued element (2.87, SD 0.566) when compared to care quality (3.75, SD 0.649) and the support given by relationships within the care context (3.91, SD 0.274).

Aim 2: What facilitates the management of cancer patients’ condition?

From the analysis of the contents which arose during the focus group sessions, we observed that for each main theme, several aspects emerged to describe it. Patients reported how these aspects have a direct influence on the effectiveness of their management of cancer experience.

Each code was linked to a more general theme, as shown in Table 2.

### Care quality

Participants reported three main elements that express the quality of the care process they follow: the clarity and availability of explanations provided by the caring team, professional competence and research advancement and the amount of communication between different healthcare professionals about each patient.

### Knowledge

Patients reported that one of the most important things within the care process is the feeling of knowing and understanding what is happening to them. Participants reported that, in order to deal with this, it is fundamental that doctors take time to explain their decisions and the consequences of those decisions in order to present an informed choice.

Knowledge is important…it’s really important …because you put yourself in the hands [of the doctor] but you don’t know, you don’t know how to move, you just don’t know. I like my doctor because she’s always there; if I need anything I simply have to call her

Patients often recognise that they do not have the ‘technical’ knowledge of their doctors and they recognise that it is essential for them to stay in touch with their oncologist…

I’m ignorant, I didn’t go to college or finish high school, so if they told me things so that I could understand them more… I always ask it blatantly… ‘I don’t get it’… until now, they have always answered…

It’s important for me to have the ability to have telephone contact with my doctor

…so that physicians can explain everything that is not clear about diagnosis and treatment.

My oncologists and I, before doing anything have always spoken, they explained to me everything and we compared our views, I have always been sincere…

I have been following the therapy for 3 months and kept receiving email for all sorts of lab reports and [the doctor] told me: ‘we are not doing the chemotherapy […] we are shifting to immunotherapy’, like that, no explanation given, nothing, zero, he just hands me my box and says: ‘you’ll start taking these’. If he informed me, instead, if you take these you’ll have these kinds of side effects, I can make a choice.

### Competence and research advance

Another critical feature of a quality care process is the competence and research advance of the care team. In order to feel that a care process is trustworthy, patients reported the importance of trust both in the single clinician and in the treatment protocol itself.

Research is everything, it was chemo [therapy] 2 years ago, now it’s immunotherapy (…) maybe in 2 years, who knows, a Martian will cure you by hand imposing (…) if they lead to results

In this regard, patients report the desire to have information about the most innovative treatment plans, in a useful and quick way.

I believe that it is important to have information on more innovative care

It’s important that [clinicians] behave professionally, that people do their job with heart

It’s important to decide your care process swiftly

### Communication between different healthcare professionals

Finally, an additional element valued by patients in assessing the care quality is the level of communication between different specialists that are in charge of the care process of each patient.

I think it is better to have a team of people that decide what is best for me [instead of a single doctor], period.

I need the certainty that the team talks, according to different shift works, so that any clinician who is currently on duty knows my clinical history

It is very important for me to know that I can always contact the doctors who operated on me if I need them or if I have concerns about something that is happening to me

Patients’ perception of care quality was revealed to encompass different components. The competence and ability to treat their condition is, as expected, one of these, but along with those communication played an important role as well. Patients reported that communication between different healthcare professionals is valued in order to feel that the whole team of care providers are working to help the patient. Finally, a fundamental element is the ability for the staff to provide clear and adequate information to patients about their condition, the status and planning of their treatment and the motivations that lie behind important clinical decisions.

### Relationships in the care context

The most valued element in the management of the patient condition, though, is the relationship. This factor was reported to be fundamental in three different aspects: the relationship with healthcare professionals, the assistance of family members and the ability to receive psychological support, in either an individual or group setting.

### A therapeutic empathy

The most valued element, when dealing with cancer treatment, was the presence of an empathic relationship. Specifically, patients reported the importance of a relationship with the clinician not just limited to the communication of medical information but also encompassing an amount of ‘therapeutic empathy’ that provides a ‘humane’ interface that the patient can interact with.

It’s been a month now, she [the clinician] calls me twice or three times every week and asks me how I feel, the other [doctor] never showed interest (…) the greatest fortune is to have a doctor [like her] to count on

The most important thing, in my opinion, is to have a relationship with the oncologist … this is the first thing, therapy comes second … the oncologist is a clinician, but he also has to be a friend

When I talk about empathy, I don’t mean pleasantness, I refer to a therapeutic empathy so that you find a doctor that makes you feel comfortable while discussing all the options (…) that can give you some humane support, some practical support. (…) I believe that healthcare staff (…) should follow a common line tailored to a specific individual, for you, for me, for anyoneI

believe that is important for clinicians to be more humane with patients because not all the doctors treat you like they should.

It’s important that clinicians are available to listen to the patient’s need (…) [they] should pay attention not to humiliate, even accidentally, the patients.

The team should have patience and comfort towards the patients

### Family members’ support

Another important element of support during the care process is the close presence of family members.

I managed to deal with it, to accept it, to go on… as I said, my family granted me the strength necessary

It’s hard for those who do not have a family by their side (…) it’s important to have them close

### Professional psychological support

Finally, the importance of psychological support, from individual, couple and group settings—as evidenced in other studies [40]—is often reported as necessary, especially during the hardest phases of the diagnosis and care process.

[In some moments] it would be better to have psychological support more than medical support that, in the end, tells you ‘the CT scan is OK, the mass reduced, increased, there is a new metastasis…

I think this [group meeting] it’s a stimulus to be more involved, not that this changes anything, but to have some kind of exchange. For instance, I meet people with my same problem

When facing a critical situation, like receiving a cancer diagnosis and undertaking treatment, the support provided by people around them is highly valued by patients. The importance of empathy, patience and humane support from the care team was sometimes reported as even higher than that of the therapy itself. The feeling of being listened to by clinicians is reported as necessary when facing important treatment decisions and when information is communicated. When patients have to deal with their condition outside of the clinical context, a great deal of importance is given to family support and to having someone who will accompany the patients in the most difficult moments, from the communication of the disease to the burden of daily life implementation of treatments. Patients reported the importance of professional psychological support to help them understand and overcome the emotional distress related to having cancer.

### Direct control

Participants expressed opinions regarding the ability to exert direct control over two elements of their care process: clinical decision-making and treatment management.

### Perception of direct control

When expressing their opinions about direct choice of their treatment, patients presented conflicted points of view. While they valued the ability to select between treatment options, patients also acknowledged the inability to make a complete informed decision because of their lack of clinical competence. Hence, their choice is necessarily influenced by the selection of options provided by the clinician. They also expressed the importance of other people being involved in the choice more than the choice itself and claimed that they were the one who intentionally retired from the decision in favour of the doctor’s opinion.

That was my choice because I chose it (…) and I opted for it because of the fact that you can slow the treatment down if you are too tired

You get to a point where you are not the one who makes the decisions, it depends on what others tell you, it depends on your condition, it depends totally on the people you meet, I mean on the doctor you meet (…) on the ability of the clinician to understand you

I asked her [the clinician], ‘what would have you done in my shoes?’ … at this point, I had to say this because I’m not able to make a decision (…) so I trusted the clinician.

The doctor knows why I chose this or that drug, so (…) clinicians are the first who route the choice; they know that I chose this one because I had no other choice, that he [the clinician] chose it because it was the best option. So clinicians are more aware of us (…) none of us chose a drug choice that no one offered us, that no clinician offered us.

At a certain point, I just stepped back, just for the sake of choice … I said I’m suffering … I won’t say, I won’t do (…) sometimes you just lose contact with reality

### Managing treatment schedule

Another element that emerged about the importance of having direct control over their treatment is the ability to manage everyday life. Patients valued the ability to choose and organise the treatment schedule according to their life rhythm.

I take the drug every day, I skip weekends, to let my body rest, it makes you sleep, so I’m glad I can take it before going to sleep so that I can live my life outside of those 2–3 hours.

Patients’ opinions about the control of their condition were slightly different from what the literature anticipates. While control over their disease is still an important element to help them manage their condition, the expression of this control does not focus on the clinical decision made by clinicians. Patients reported to be aware of the lack of specific knowledge required to make important medical decisions directly and they tend to trust their clinicians instead of controlling the direction of specific decisions. They, instead, value control when dealing with the daily management of the treatment, especially in the follow-up of the treatment when they are back home.

### Choice of the care team

When prompting patients to express their opinion about elements that helped them manage their condition, an original element emerged in all three groups. Several participants expressed that the dimension that allowed them to perceive to have control over what was happening to them was the awareness of having the power to choose or change the doctor, the care team or the health organisation that was taking care of them. This ability was reported as one of the main choices completely entitled to them as patients’ choice of their caretaker since it has a direct influence on the future of their care process.

It’s a matter of choice. The choice here is not to pick a vaccine that you simply choose the one that gives you less side effects (…) this is completely different. The only time when I really faltered, and I opted for hearing another opinion, the [former] surgeon told me that [the tumour] was not operable (…) and then I started to think ‘what should I do?’ since I already followed the whole radiotherapy course. (…) but then I chose to get the operation and to seek another opinion (…) I trusted a surgeon I felt empathic

I had the feeling, when I arrived here, that if there is any chance of survival this is the place where I could get it. (…) There are hospitals that I have been to where [I thought] ‘Ok, what have I done, I’m going to die for sure here

This commonly reported element provided insight to a dimension in which patients feel they have a direct and un-modulated control. This choice was reported as strictly related to the other dimensions emerging during focus group sessions. Patients’ decision to change the healthcare staff could be due to problems related to the relationship with the clinicians or, in general, with the lack of empathy with the rest of the staff. Other reasons to change provider of care could be related to the quality of care perceived by patients in terms of information communication or advancement of the treatment.

## Discussion

When facing cancer, being able to adopt an active stance is a fundamental element that allows patients to better manage their condition. Empowerment, in this perspective, is often defined as an increased control over the care process in the treatment definition process. From the clinician’s perspective, this often translates as the patients picking a treatment option among those presented by the clinician. However, patient contributions on this topic showed that other elements in the care process enable an improved management of their condition.

Empowerment, in the wider scope of a process that enhances patient management, was revealed to encompass different elements and actions other than medical decision making.

The element that was most valued by the participants was the relational component of the care process. As commonly expressed in the literature [41], this primarily translates with an empathic approach by clinicians that leaves room for patient’s doubts and worries in order to provide a better understanding of their condition and to assess potential complications and difficulties that may emerge in the therapy process.

Relational support benefits patients in the hospital setting with health care professionals, as well as in the domestic environment with informal caregivers when patients are in charge of the self-management of their treatment, symptom monitoring and maintenance of a healthy lifestyle.

Another important relational support valued by patients comes from professional psychological support, especially in the first phases of the care process that may entail fears and doubts which may disorient patients and provide an obstacle to their perceived efficacy in facing their condition [42]. The ability to receive psychological support was reported as an element that let patients perceive themselves to be considered as more than just clinical cases.

Communication within the patient–clinician relationship was also valued as an element of the quality of care. The ability to effectively inform patients and exchange information within the care team was recognised to be as valuable as research advancement and the efficacy of the care itself. In this perspective, a synergic and cooperative relationship with all members of staff makes patients feel part of the care process, even though they realise the different degree of competence and education that differentiates them from clinicians.

For this reason, patients reported that their degree of control over their condition does not focus only on a direct intervention on the clinical decisional process but implies a broader scope that begins with the choice of the care team itself. In this account, the ability to switch from their physician who is currently taking care of them to a new team of health care professionals, that they feel more empathic and trustworthy, makes them feel in control of their care process. Choosing who is going to make decisions for their treatment is actually the first decision that impacts their care.

The most common definition of empowerment frames its major benefit as an enhancement of patient–clinician relationship [43]. Patient information, though, is not a sufficient factor to guarantee cooperation with the caretaker. An informed patient may, in some cases, disagree with the clinician’s treatment plan or decide to make tweaks and changes to the daily schedule. From this perspective, the choice to adhere to a care plan proposed by the physician is highly influenced by the choice of the physician itself. Deciding to delegate and follow the decision plan made by someone else does not necessarily mean that the patient is not empowered. On the contrary, it may be the consequence of a very direct and first-hand choice regarding the care plan [29]. The evaluation that patients carry out in order to make decisions cannot rely on a technical competence that they do not possess, but it may rely on a relational and emotional competence that allows the patient to have information about how much they can trust the person who is making the calls for them [53]. The relationship with the clinical team could have a modulating effect on compliance and adherence which is independent from the literacy or information level. In this perspective, the relational and clinical level of care of the patient cannot be differentiated.

In this sense, patients consider both the quality of care and of the care relationship. Competence and the relationship taken individually do not seem to be sufficient to predict patients’ choice of the caring team. This means that taking care only of the disease, not considering the patient’s preferences and emotional response, is not enough in the same way in which caring only for the relationship, avoiding the sharing of unpleasant information, is not enough.

Thinking about empowerment just as a process aimed to increase patient’s direct control or literacy [44] does not fully consider their involvement in the care plan and cooperation with health care providers.

### Limitations and future directions

Our study suffered from some important limitations that have to be considered in order to correctly understand the extent of our considerations.

Firstly, it must be considered that the sample was composed almost completely of women and the majority of the participants suffered from breast cancer. The specificity of this sample could express the needs and perception of a specific population and this could have implications on the generalisability of our results.

A second element that must be considered is that all participants were recruited by the psychological support service of one single institute. The sample was recruited among people who were either receiving long-term psychological assistance or undergoing psychological assessment. People that decided to avail themselves of psychological assistance may be individuals who already value relational support within the care process and this could have had an influence on our results.

Another limitation relative to our sample is the small size. Even though the sample is reasonable for a qualitative focus-group study, the number of people recruited influences the generalisability.

For these reasons, we think that it would be very interesting in the future to investigate patients’ perception of empowerment on a wider and more heterogeneous sample to capture differences that could be relevant.

The other limitation that must be considered for this study is the absence of a standardised measure. In order to investigate these themes with a greater reliability, a future direction of this study is to confirm our results adopting a quantitative approach.

Despite these limitations, our study provides an original overview of facilitators to empowerment from the patients’ perspective and may contribute a new angle to the existing literature on patient empowerment. Future directions of our work will focus on a further exploration of patients’ perception of empowerment in a quantitative stance and with a bigger and more balanced sample to confirm what we observed and increase the relevance of our results.

## Conclusions

Patient reporting on empowerment’s enablers provided a valuable perspective on a complex and multi-faced concept. Revising the definition of empowerment from a non-clinical point of view stressed the importance of elements that may not always be implemented in the actual care process.

The consistency and clarity of these results support the hypothesis that a deeper consideration of the relational needs of the patient could lead to a better care practice and a stronger cooperation between patients and health care providers. A cooperative stance can have positive effects in terms of self-management and compliance to the treatment plan. We think that, in order to foster actual empowerment, patients’ perspective on what and what is not is up to them has to be considered.

Future researchers should investigate with a wider sample the effect of the themes identified by patients as highly relevant in different contexts of the care process.

## Conflicts of interest

The authors declare that they have no conflict of interest.

## Funding statement

The authors did not receive any funding for this work.

## Figures and Tables

**Table 1.** Focus group participants’ demographic, cancer and treatment characteristics.

N (% female)		33 (97%)
Age (mean (SD); Range min–max)		53 (10, 97); 30–80
Cancer type (N(%))	Breast	21 (62%)
	Ovarian	4 (11%)
	Uterine	5 (15%)
	Prostate	1 (3%)
	Sarcoma	1 (3%)
	Urogenital	1 (3%)
	Bladder	1 (3%)

**Table 2.** Codes representative of specific domains for each theme.

Theme	Codes
1. Care quality	Knowledge
	Competence and research advance
	Communication between different healthcare professionals
2. Relationship in the care context	Therapeutic empathy
	Family members support
	Professional psychological support
3. Perception of direct control	Control over treatment decisions
	Managing treatment schedule
	Choice of the care team
